# Perceptions of HIV/AIDS in One’s Community Predict HIV Testing

**DOI:** 10.1007/s10461-012-0243-9

**Published:** 2012-07-06

**Authors:** Lu Shi, David Kanouse, Susie Baldwin, Junyeop Kim

**Affiliations:** 1 Department of Health Services, UCLA School of Public Health, 650 Charles E. Young Drive S. 61-253 CHS, Los Angeles, CA 90095 USA; 2 RAND Corporation, Santa Monica, CA USA; 3 Department of Public Health, LA County, Los Angeles, CA USA; 4 Department of Education, Hongik University, Seoul, South Korea

**Keywords:** HIV, Early detection, HIV test, Perceived seriousness, Los Angeles, Propensity score

## Abstract

Using a subsample of respondents to the 2005 Los Angeles County health survey, we examined the relationship between perceptions of the seriousness of HIV/AIDS in one’s community and HIV testing. We constructed a propensity score-based matched sample of three groups with differing perceptions of the seriousness of HIV in their community: high perceived seriousness, low perceived seriousness, and uncertain about seriousness. We compared HIV testing behavior in the three groups before and after using propensity score matching to control for selection on observed covariates. The unadjusted comparison showed a testing rate of 30.2 % among those perceiving high seriousness, 11.4 percentage points higher than the 18.8 % testing rate among those perceiving low seriousness. After propensity score matching, the adjusted testing difference was 7.0 percentage points (*p* < 0.05). Those uncertain about the seriousness of HIV did not differ significantly in their testing behavior from those perceiving high seriousness.

## Resumen

Utilizando una submuestra de los entrevistados de la Encuesta de Salud del Condado de Los Ángeles 2005, se examinó la relación entre su percepción de la gravedad del VIH/SIDA en sus comunidades y el sometimiento a la prueba del VIH. Con base en medidas de propensión coincidente, se generó una muestra de tres grupos con distinta percepción de la gravedad del VIH en sus comunidades: percepción de alta gravedad, percepción de poca gravedad e indecisión sobre la gravedad. Se comparó el comportamiento del sometimiento a la prueba del VIH en los tres grupos, antes y después de utilizar el método de medidas de propensión coincidente PSM (por sus siglas en inglés, *propensity score matching*), para controlar la selección de las covariables observadas. La comparación no ajustada mostró una tasa de sometimiento a la prueba del 30.2 % entre los que percibían el VIH de alta gravedad, 11.4 puntos porcentuales mayor que la tasa de sometimiento a la prueba del 18.8 % entre los que lo percibían de poca gravedad. Después del PSM, la diferencia ajustada de sometimiento a la prueba fue de 7.0 puntos porcentuales (*p* < 0.05). El comportamiento de sometimiento a la prueba entre aquéllos indecisos sobre la gravedad del VIH no difirió significativamente del de los que lo percibían de alta gravedad.

## Introduction

It is well established that perceived personal vulnerability to health threats can motivate self-protective behaviors [1, 2]. For health threats that are related to personal risk behaviors, such as smoking or engagement in risky sex, risk perceptions are related both to the individual’s knowledge of having engaged in potentially risky behavior and to beliefs that such behaviors will lead to a health problem.

In the case of behaviors that could potentially expose people to infectious diseases, Kalichman and Cain [3] have argued that people have an understanding as “intuitive epidemiologists” that their risk of becoming infected is directly related to the prevalence of disease in the community. This intuitive understanding, combined with the motive to avoid health threats, would lead us to expect a relationship between perceived prevalence of disease in the community and engagement in risky or self-protective behaviors [4]. Consistent with that expectation, Kalichman and Cain [3] found that among men and women receiving diagnostic and treatment services at a sexually transmitted infections (STI) clinic, those who estimated a lower AIDS burden in their city relative to other cities reported having greater numbers of sex partners, higher rates of risky sexual practices, higher rates of STI, and lower rates of HIV testing compared with those who estimated an average or higher AIDS burden in their city relative to other cities. These results suggest that perceptions of the prevalence of HIV and other STIs in the community can affect both willingness to engage in risky sexual behaviors and motivation to be tested for HIV. Such an association could have important implications for the design of public health messages to reduce risky sexual behavior and encourage HIV testing.

Several studies have shown linkages between perceived personal risk of HIV infection and HIV testing [5–8]. However, few previous studies have examined the relationship between perceived community vulnerability to HIV/AIDS and either risky sexual behavior or HIV testing. Downing et al. [5] interviewed 66 injection drug users in three counties in the San Francisco Bay area and reported that many cited the high prevalence of HIV in their communities rather than their own risk behavior as a reason they were at risk for HIV. Klepinger et al. [9] investigated whether perceptions of the local prevalence of disease serve as a predictor of health behavior, particularly behaviors associated with infectious diseases such as HIV/AIDS. In their study, men and women who received diagnostic and treatment services at an STI clinic completed anonymous surveys of perceived prevalence of HIV/AIDS and other STI and of sexual risk and self-protective behaviors. Participants who estimated a lower AIDS burden in their city relative to other US cities demonstrated greater numbers of sex partners, higher rates of sexual risk practices, and higher rates of STI. They were also less likely to have been tested for HIV. With these findings, Klepinger et al. [9] concluded that among STD clinic patients the perceived local disease prevalence may predict sexual risk behaviors. As a further step, Ahituv et al. [10] used the National Longitudinal Survey of Youth (since 1979) to show that use of condoms is related to the prevalence of AIDS in one’s state of residence. A 1 % increase in AIDS increases condom use significantly, up to 50 % for the groups most responsive to prevalence (men, urban residents, single).

Despite these findings, Poppen and Reisen [11] concluded that the research literature had generally failed to demonstrate a link between risk perception and self-protective behavior, citing validity issues (e.g., the monolithic definition of condom use as the only measure of safe sex, and the questionable use of recalled risk behavior that had occurred more than a week ago) and the lack of control variables (e.g., variables about the actual relationship with the partner) as major limitations on the ability to draw conclusions from this literature.

Although an association between HIV/AIDS risk-related behavior and perceptions of HIV/AIDS risk at the community level has been found in several empirical studies, the evidence remains inadequate to establish an independent link between perceived community vulnerability and individual risk behaviors. Even less is known about the association between perceived community vulnerability and HIV testing behavior. To fill this research gap, this paper aims to determine the factors that influence perceptions of how seriously HIV/AIDS affects one’s community and to examine whether perceived seriousness of HIV/AIDS in one’s community influences HIV testing behavior.

We examine the relationship between perceptions of community risk for HIV/AIDS and HIV testing in a representative community sample of adult residents of Los Angeles County. We use a propensity matching approach [12]. This approach controls for self-selection on the characteristic of interest (high or low perceived seriousness of HIV in the community) by calculating propensity scores and using them to balance the subsamples for comparison in a way that automatically controls for selection on observed covariates. By constructing matched sample based on propensity score, we approximate an experimental setting in which people are randomly assigned to high or low risk perception of AIDS risk and compare their HIV testing behavior therewith. Thus, this technique provides a substantial improvement over multiple regression since the latter is subject to significant selection bias.

## Methods

### Key Variables: The Outcome and the Exposure

The 2005 Los Angeles County health survey (LACHS) used random-digit dial methods to conduct telephone interviews with 8648 adult residents of Los Angeles County ages 18 and older regarding their health and health-related needs. The survey was conducted between January and June 2005 and obtained a cooperation rate of 49 % and a response rate of 23 %. A random subsample of 909 adults completed a supplemental HIV/AIDS questionnaire. All 909 subjects selected to answer the HIV/AIDS questionnaire were asked the question “in your opinion, how serious of a health issue is HIV/AIDS in your community—very serious, somewhat serious, not too serious or not at all serious?” A significant proportion of this subsample (102 out of 909) chose “don’t know.” For our analysis of whether low perceived vulnerability for HIV/AIDS is associated with HIV testing behavior, we recoded this questionnaire item into two binary variables: one that denotes low perceived vulnerability (1 for those who responded “not at all serious” or “not too serious”, and 0 for those who responded “very serious,” “serious,” or “don’t know”) and one that denotes uncertainty (1 for those who responded “don’t know” and 0 for those who responded otherwise).

In our subsample, there were 30 participants (13.3 % of all HIV testers) who reported that they took the HIV test because the test was mandatory for them. As this group’s testing behavior might not have been affected by their perception of community vulnerability, we excluded them from our analysis. The final sample size for our analysis is 843 due to the issue of missing values in predictors.

### Analysis Strategy

In order to reduce the selection bias that can occur in a nonexperimental study, we use propensity score matching to “reconstruct” a sample that mimics the results of the random assignment component in a randomized clinical trial, by creating a control group that has similar values on observed confounders in the exposure group and differs only with respect to an “exposure variable” of interest [13]. Propensity score is defined as the subject’s predicted probability of receiving the treatment/exposure. In our case, the predicted probability of perceiving low vulnerability, as estimated from a probit regression, is the propensity score for hypothesis I, and the predicted probability of expressing uncertainty is the propensity score for hypothesis II. After each individual is assigned a specific propensity score, each individual in “the exposure group” is then compared with “control group” members that have a close propensity score, and their differences in the outcome variable (in our case, HIV testing) are summed to give an overall difference that indicates the exposure variable’s independent association with the outcome variable.

For our analysis, we conducted propensity score matching to compare the HIV testing difference between the group perceiving low vulnerability for HIV and the group perceiving high vulnerability for HIV, as well as the testing difference between the group expressing uncertainty and the group perceiving high vulnerability. In selecting probit regression predictors for generating the propensity score, we chose to include health insurance coverage and household income status in our predictor list in addition to socio-demographic covariates (age, gender/sexual orientation, race/ethnicity and education), since health insurance coverage and household income status have been shown to be associated with HIV testing behavior [14–16]. For both comparisons, *t* tests were performed to confirm that the matching procedures resulted in groups that were similar with respect to each confounding variable.

When implementing the propensity score matching for our two comparisons, we use kernel-based matching as developed in STATA package ATTK [17]. With kernel-based matching, each exposure case is compared with a weighted sum of all control cases, with the weights inversely related to the propensity score difference between the exposure case and the control case. We chose this method from a variety of propensity score matching algorithms because kernel-based matching ensures that all cases in the control group will be used, whereas some other matching algorithms throw away control cases whose propensity scores are not similar to any of the treatment cases [17]. For both our pairwise comparisons, the sample has less than one thousand observations, and therefore losing a substantial proportion of this already small sample could hurt the robustness of the model results.

## Results

### Descriptive Statistics

When asked about whether an HIV test had been taken in the past 2 years, 226 of the 843 subjects (26.8 %) answered yes. When rating the seriousness of HIV epidemic in their community on the Likert scale, 80 (9.5 %) said “not at all serious,” 112 (13.3 %) said “not too serious,” 184 (21.8 %) said “somewhat serious,” and 373 (44.3 %) said “very serious.” Table 1 lists descriptive statistics for variables used in the propensity score matching, with the sample of 843 respondents who answered the question of HIV testing. We can see that some of the subcategories have relatively small counts, like the gay men (*n* = 23). And therefore any significant effect on the outcome, as shown by this subcategory, should be interpreted with caution.

**Table 1:** Descriptive statistics about variables used in the propensity score matching

Variables	Descriptive statistics
Outcome variable	
Tested for HIV during the past 2 years	
Tested	226 (26.8 %)
Not tested	617 (73.2 %)
Exposure variable	
Seriousness of HIV/AIDS epidemic in one’s community	
“Not at all serious” or “not very serious”	192 (22.8 %)
“Don’t know”	94 (11.1 %)
“Somewhat serious” or “very serious”	557 (66.1 %)
Predictors	
Age (mean)	47.5 (0.6)
Education	
Less than high school (referent group)	172 (20.4 %)
High school only	168 (19.9 %)
Some college	181 (21.5 %)
Graduated from college	322 (38.2 %)
Gender/Sexual orientation	
Female (referent group)	434 (51.5 %)
Non-gay men	386 (45.8 %)
Gay men	23 (2.7 %)
Race/ethnicity	
Black (referent group)	64 (7.6 %)
Hispanic	349 (41.4 %)
White	344 (40.8 %)
Asian	86 (10.2 %)
Continuous health insurance past 12 months	
No	204 (24.2 %)
Yes	639 (75.8 %)
Household (HH) income	
Below federal poverty level^a^	156 (18.5 %)
At or above federal poverty level	687(81.5 %)
*N* = *843*	

^a^Based on US Census 2003 federal poverty level (FPL) thresholds which for a family of four (two adult, two dependents) correspond to annual incomes of $18,700 (100 % FPL), $37,300 (200 % FPL), and $56,000 (300 % FPL). These thresholds were the values at the time of survey interviewing

#### Estimation of the Propensity Score

Table 2 provides the unadjusted associations between perceived seriousness of HIV/AIDS as a health issue and demographic covariates. An ANOVA test of age by perceived HIV/AIDS seriousness categories shows that age differs significantly across these categories (*F* = 6.19, *p* = 0.0021), and those perceiving high HIV/AIDS seriousness for their community appear to be younger than the other two groups (μ = 46.1, SE = 0.69). Results of the probit regressions used to estimate the propensity scores are presented in Table 3. The probit regression that examines the contrast between high and low perceived seriousness shows that compared with women non-gay men are more likely to perceive low seriousness for HIV (probit coefficient = 0.20, *z* = 2.00, *p* = 0.046) and gay men are less likely to perceive low seriousness for HIV (probit coefficient = −0.90, z = −1.95, *p* = 0.051). The probit regression that examines the contrast between high perceived seriousness and uncertainty about seriousness yields no significant predictors.

**Table 2:** Association between perceived seriousness and demographic covariates (unadjusted)

	Low perceived seriousness	Uncertain	High perceived seriousness	χ2	*p*
	Frequency/percent	Frequency/percent	Frequency/percent		
Gender/sexual orientation					
Female	90/19.69	53/11.60	314/68.71	9.293	0.010
Male non-gay	102/26.42	41/10.62	243/62.95		
Male gay	1/4.35	0/0.00	22/95.65		
Race/ethnicity					
Latino	76/21.78	39/11.17	234/67.05	11.05	0.087
African American	10/15.63	8/12.50	46/71.88		
White	82/23.84	31/9.01	231/67.15		
Asian	24/27.91	16/18.60	46/53.49		
Education					
Less than high school	36/20.93	26/15.12	110/63.95	5.262	0.511
High school	39/23.21	21/12.50	108/64.29		
Some college	45/24.86	16/8.84	120/66.30		
College graduate	72/22.36	31/9.63	219/68.01		
Continuous health insurance coverage during past 12 months					
Yes	154/24.10	74/11.58	411/64.32	3.701	0.157
No	38/18.63	20/9.80	146/71.57		
HH Income below federal poverty level					
Yes	162/23.58	74/10.77	451/65.65	1.637	0.441
No	30/19.23	20/12.82	106/67.95		

	Mean/SE	Mean/SE	Mean/SE	*F*	*p*
Age	49.5/1.30	51.4/2.00	46.1/0.69	6.19	0.0021

**Table 3:** Two probit regressions predicting the propensity scores of low perceived seriousness and feeling uncertain about the seriousness of HIV in one’s community

	Low perceived seriousness			Uncertainty		
	Coefficient	*z*	*p*	Coefficient	*z*	*p*
Age	−0.01	−0.80	0.421	−0.02	−0.94	0.349
Age squared	0.00	1.23	0.218	0.00	1.51	0.130
Gender/sexual orientation (ref. female)						
Heterosexual men	0.20**	2.00	0.046	−0.03	−0.22	0.829
Gay men	−0.90*	−1.95	0.051	Dropped^a^		
Race/ethnicity (ref. African American)						
Latino	0.40*	1.73	0.083	0.01	0.06	0.955
Non-Latino White	0.33	0.33	0.147	−0.18	−0.69	0.488
Asian	0.55**	2.08	0.037	0.42	1.45	0.147
Education (ref. not finished high school)						
Graduated from high school	0.05	0.26	0.797	−0.12	−0.60	0.549
Some college	0.11	0.65	0.518	−0.30	−1.49	0.135
College graduate	−0.05	−0.26	0.798	−0.29	−1.53	0.126
Continuous health insurance coverage during past 12 months	0.20	1.55	0.122	0.09	0.56	0.574
HH income below federal poverty level	−0.06	−0.37	0.708	0.06	0.34	0.731

*Source* 2005 Los Angeles County health survey. Coefficient significant at * 10 %, ** 5 %, and *** 1 %

^a^No one in the male and gay group felt uncertain about community seriousness of HIV

The propensity scores obtained through this method successfully balanced the study samples. Because none of the covariate differences between “exposure group” and “control group” are statistically significant, propensity score matching is an appropriate method to adopt and we do not have to adjust for the covariate effect in the second step of comparing testing rates.

#### Comparison Between the Matched Samples

Table 4 shows the results of unadjusted comparisons and the two propensity score matched comparisons. The unadjusted comparison shows a testing rate of 30.2 % among those perceiving high seriousness of HIV in their community, 11.4 percentage points higher than the 18.8 % testing rate among those perceiving low seriousness (*z* = −3.06, *p* = 0.002). Kernel-based propensity score matching adjusts the testing difference to be 7.0 percentage points (*t* = −2.33, *p* = 0.010). For the comparison between the group who answered “don’t know” to the question about the seriousness of the epidemic and the group perceiving seriousness to be high, neither approach yields a statistically significant difference (unadjusted: *z* = −1.33, *p* = 0.183; propensity score adjusted: *t* = −0.84, *p* = 0.200).

**Table 4:** Contrasts of three groups’ HIV testing behaviour using unadjusted comparison and kernel-based propensity score matching

	Number of people who perceived low seriousness	Number of people who perceived high seriousness	Propensity score-adjusted proportional difference	*t*	*p*	Proportional difference unadjusted	*z*	*p*
Those perceiving low seriousness vs those perceiving high seriousness	192	557	−0.070 (0.029)**	−2.33	0.010	−0.114 (0.034)**	−3.06	0.002
Those perceiving uncertainty vs those perceiving high seriousness	94	557	−0.042 (0.050)	−0.84	0.200	−0.068 (0.048)	−1.33	0.183

*Source* 2005 Los Angeles County health survey

** *p* < 0.05

## Discussion

Results of this study are broadly consistent with previous findings by Kalichman and Cain [3] and Klepinger et al. [9] suggesting that when people perceive HIV as prevalent in their community, they are more likely to engage in protective behavior such as HIV testing. In Kalichman and Cain’s study, people who estimated a lower AIDS burden in their city relative to other US cities were less likely to have been tested for HIV [3]. These investigators also found that those who perceived a lower relative AIDS burden had a greater number of sexual partners, higher rates of both protected and unprotected vaginal and anal intercourse, and a greater number of STI diagnoses and current symptoms.

In this study, the operational measure related to HIV prevalence was perceived vulnerability of the individual’s community to HIV, as reflected in responses to the question, “In your opinion, how serious of a health issue is HIV/AIDS in your community—very serious, somewhat serious, not too serious or not at all serious?” While this question does not specifically refer to prevalence in the community, it is reasonable to interpret responses as reflecting respondents’ perceptions of how many people in the community are infected, and perhaps also of how serious are the health consequences of being infected with HIV.

Sexual orientation, gender, and race/ethnicity were all associated with perceived seriousness of HIV in the community. After balancing the sample for these and other variables, perceived seriousness of HIV in the community was strongly associated with testing behavior among Los Angeles County residents. The more serious a health issue residents perceived HIV to be, the more likely they were to report having been tested.

This study offers two methodological advantages compared with previous studies. First, these analyses are based on a probability sample of Los Angeles County adult residents and therefore may be more readily generalized to a larger population than results of previous studies on this topic. Second, the use of propensity score matching to estimate the relationship between testing behavior and perceived community vulnerability provides some of the advantages of a controlled trial, in that it allows for comparisons in which differences in other measured characteristics are controlled. This methodology reduces the bias from group differences more effectively than can be done with more traditional methods, such as adjusting for covariates using linear regression [18], allowing us to establish whether perceived community vulnerability to HIV is independently associated with HIV testing behavior.

Our results indicate that perceived community vulnerability to HIV is independently associated with HIV testing behavior. However, this demonstrated independence is only with respect to other variables that were measured in the survey and included in the propensity score matching procedure. Unlike randomization, which results in expected equivalence on all variables, both measured and unmeasured, the effectiveness of propensity matching depends on how comprehensively potentially biasing covariates have been assessed.

This study has several limitations. First, only 23 % of eligible phone numbers in the sample resulted in completed survey interviews, and only 49 % of those successfully contacted for the survey chose to participate. While these response and cooperation rates are low, they are similar to rates obtained in other local and national telephone surveys of the general population. Also importantly, the unweighted LACHS sample closely reflected the population makeup of the county’s non-institutionalized adults.

Another limitation of our study is that the LACHS was aimed only at members of households with telephones, excluding members of certain groups at increased risk of HIV, such as the homeless and incarcerated. HIV testing was measured by self-report, and is subject to errors of recall and possible response bias if respondents were motivated to give socially desirable answers. Also, while we were able to examine perceived community vulnerability to HIV, it was not possible to concurrently examine the effects of this variable while controlling for the perception of personal vulnerability, a variable that was not measured by the survey. Finally, in a cross-sectional survey with retrospectively reported behavior, it is not possible to determine the temporal ordering or causal relationship between perceptions and HIV testing behavior. While there are theoretical reasons to predict that perceived community vulnerability to HIV should increase motivation to be tested, self-perception theory [19] also suggests that people who have (have not) been tested might infer from their own behavior that HIV is (is not) a serious health issue in their community. For example, those who get tested for HIV may in effect tell themselves, “HIV must be a fairly serious problem in the community or else I wouldn’t have taken the trouble to get tested.” Moreover, most people would have received some form of counseling when they get tested for HIV, and this counseling could have increased their perception that HIV is a serious problem in the community. Thus, future research prospectively examining the relationship between perceived seriousness of HIV in the community and HIV testing is needed to shed further light on causal direction.

A third possible limitation of our study is that the LACHS used a window period of “the past 2 years” to measure HIV testing behavior. The CDC recommends annual testing for people with HIV risk factors and routine HIV screening in health care settings (such as at medical check-ups) for other people aged 13–64 who do not have HIV risk factors. Thus, the way LACHS measures HIV testing might not reflect whether the individual is being tested for HIV with recommended frequency.

From a public health perspective, perceptions of HIV in the community may hold important implications for HIV prevention and control. Our findings suggest that perceiving the epidemic as a serious issue in one’s community serves as an independent motivating factor for protective behavior, in the form of HIV testing. If protective behavior can be influenced by public health messages or other interventions, then it may be possible to bolster HIV prevention efforts in high-prevalence areas by emphasizing HIV levels in the community as a reason to reduce risk behavior and seek periodic testing. Since HIV prevalence is an external risk factor for which individuals are unlikely to feel personally responsible, they may be less likely to defensively minimize HIV risk or reject the importance of testing than they are when the source of risk is tied more directly to their own behavior [20, 21].

Current CDC guidelines call for routine, voluntary HIV testing of all persons aged 13–64 in health care settings [22]. Efforts to promote HIV testing at the community level that make their appeal based on community HIV prevalence are therefore consistent with current CDC guidelines and with the rationale behind them, which is that previous efforts to promote testing based on individual risk behavior were missing too many HIV-positive people. Appeals based on community prevalence may help to destigmatize HIV testing by weakening the presumption that HIV test-seeking reflects stigmatized risk behavior or membership in a stigmatized group. Such messages may also help to counteract the decline since the later 1980s in the perception of HIV as an urgent health problem in the US [23] and the parallel decline in personal concern about becoming infected with HIV [24]. Responses to messages about the prevalence and seriousness of HIV at the community level are a worthy topic for future research.
